# Dairy Cattle Infection with Bovine Rotavirus at Different Growth Stages and Its Impact on Health and Productivity

**DOI:** 10.3390/ani15111628

**Published:** 2025-06-01

**Authors:** Xinfeng Hou, Zheng Niu, Shengru Wu, Qian Du, Guanglei Liu, Lichen Nie, Changlei Zhu, Yudong Qiu, Yong Huang, Yangchun Cao, Dewen Tong

**Affiliations:** 1 College of Veterinary Medicine, Northwest A&F University, Xianyang 712100, China; houxinfeng@jlbry.com (X.H.); nz0511@126.com (Z.N.); dewey3600@nwsuaf.edu.cn (Q.D.); nilichen2018@163.com (L.N.); 18236718488@163.com (C.Z.); 15891777296@163.com (Y.Q.); yonghuang@nwsuaf.edu.cn (Y.H.); 2 JUNLEBAO-Northwest A&F University Cooperation Dairy Research Institute, Leyuan Animal Husbandry, JUNLEBAO Company, Shijiazhuang 050200, China; guanglei1979@126.com; 3 College of Animal Science and Technology, Northwest A&F University, Xianyang 712100, China; wushengru2013@nwafu.edu.cn; 4 Key Laboratory of Ruminant Disease Prevention and Control (West), Ministry of Agriculture and Rural Affairs, Xianyang 712100, China; 5 Engineering Research Center of Efficient New Vaccines for Animals, Universities of Shaanxi Province, Xianyang 712100, China; 6 Engineering Research Center of Efficient New Vaccines for Animals, Ministry of Education, Xianyang 712100, China

**Keywords:** bovine rotavirus (BRV), prevalence, calf health, production performance

## Abstract

This study evaluated the prevalence of BRV infection and its impact on the health of dairy cows at different growth stages, with a particular focus on milk-fed calves. BRV was detected in rectal swabs via RT–qPCR. In the total sample of 2400 dairy cows examined at three farms, 103 (4.29%) were found to be positive for BRV. Similarly, of the 480 milk-fed calves, 70 (14.58%) were also found to be positive for BRV. Negative binomial regression analysis revealed that older farms had higher average BRV positivity rates than new farms did and that the BRV positivity rate was higher in autumn than in the other three seasons. Additionally, BRV positivity rates gradually decreased as calf age increased. In milk-fed calves, BRV positivity was positively correlated with morbidity and mortality and negatively correlated with ADG and ADGPR. These findings provide valuable insights for farm management, particularly for older farms, highlighting the detrimental effects of BRV infection on milk-fed calves and underscoring the importance of targeted control strategies to reduce BRV prevalence and optimize dairy production efficiency.

## Simple Summary

As an important infectious disease, bovine rotavirus (BRV) poses a serious threat to cows, especially milk-fed calves. BRV can cause severe diarrhea, dehydration, and even death in calves. Hebei, a large dairy farming province in China, has a considerable number of dairy cows, but the population positive rate of BRV in farms of different ages during large-scale farming in this region is not known, and its effect on the production indicators of morbidity, mortality, average daily weight gain (ADG), and average daily weight gain pass rate (ADGPR) of milk-fed calves is not clear. The results showed that milk-fed calves were the most susceptible group to BRV infection, and the positive rate was positively correlated with morbidity and mortality and negatively correlated with ADG and ADGPR. Therefore, strengthening the control of BRV is very important to reduce the loss of large-scale farming.

## 1. Introduction

Production of milk, as an essential source of nutrition for humans, is projected to grow at an annual rate of 1.5% over the next decade, reaching 1.039 billion tons by 2032. This growth is expected to surpass that of most other major agricultural commodities, as reported by the Organization for Economic Co-operation and Development (OECD) and the Food and Agriculture Organization of the United Nations (FAO) [1]. The number of dairy animals is anticipated to increase by 1.3% annually, with dairy cows playing a key role in this growth [1]. Diarrhea, particularly affecting the growth and health of calves, is a significant challenge on farms. This leads to reduced growth rates, increased mortality, and higher veterinary costs, which impact overall farm productivity [2]. Diarrhea is closely associated with increased mortality and reduced growth rates, with an estimated 23% of dairy cows receiving treatment for this condition. In modern farming systems, especially where calves are commingled from different farms after stressful transportation, the incidence of this disease may rise significantly, increasing the risk of infectious diseases [3,4]. The economic burden of calf diarrhea is substantial, with losses estimated at USD 33–36 per affected calf [5,6].

Several factors have been identified as contributing to diarrhea in dairy cows, including pathogens such as viruses, bacteria, and parasites [7,8], as well as nutritional deficiencies [9,10], environmental conditions [9,11], and management practices [12]. BRV, a member of the *Eutheroviridae* family, *Rotavirus* genus [13], is considered one of the most significant viral pathogens responsible for acute diarrhea in young calves. BRV primarily affects calves up to 2 months of age, especially those between 5 and 14 days old, causing symptoms such as diarrhea, anorexia, dehydration, and depression [14]. Studies have shown that BRV infects calves at least once during their early life, with infection rates as high as 94% [3]. The reported prevalence of BRV in diarrheic calves ranges from 59% to 80% [15,16,17].

BRV primarily infects calves under 2 months of age, with infection occurring via the fecal–oral route, which negatively impacts their long-term growth [18]. Studies have shown that 27% to 36% of young calves are affected by BRV [19]. Waltner-Toews et al. (1986) reported a BRV prevalence of 18.6% across 78 dairy farms in southwestern Ontario [20]. Additionally, a recent meta-analysis in China reported a BRV prevalence ranging from 4% to 99% in calves, with the highest prevalence observed in the eastern regions, which may be related to climate and seasonal factors [21]. In summary, these findings highlight the widespread distribution of BRV and its significant impact, underscoring the need for continued attention to its control.

Although calves are considered to be a high-risk group for rotavirus diarrhea, it is equally important to assess infection rates in post-weaning and adult cattle. Firstly, it is important to note that older cattle populations may act as hidden reservoirs for rotavirus, and their persistent shedding can pose a threat to calf populations through the medium of fecal-oral transmission [22]. Secondly, following weaning, there is a decline in maternal antibodies, and the protective efficacy of vaccines is limited, resulting in an increased risk of reinfection during the immune gap period [23]. Furthermore, subclinical infections in adult cattle, despite their generally mild symptoms, have been demonstrated to result in reduced milk production and feed conversion efficiency and to exacerbate environmental viral contamination [3]. Consequently, the comprehensive monitoring of infection dynamics across diverse growth stages is of dual value for the optimization of rotavirus control strategies and the reduction of economic losses in the livestock industry.

While extensive research has been conducted on the pathogenesis and epidemiology of BRV, few studies have investigated its prevalence across different growth stages and seasons within dairy farms of varying ages. The influence of growth stage, particularly in milk-fed calves, remains underexplored, despite evidence suggesting that younger animals exhibit higher BRV positivity rates. Additionally, the relationships between BRV positivity in lactating calves and key production outcomes, such as morbidity, mortality, ADG and ADGPR, have been insufficiently studied. This study aims to fill these gaps by investigating the impact of BRV prevalence across farms, growth stages and seasons. These results will contribute to improved disease control strategies and enhanced productivity in dairy farming.

## 2. Materials and Methods

**Figure 1:**
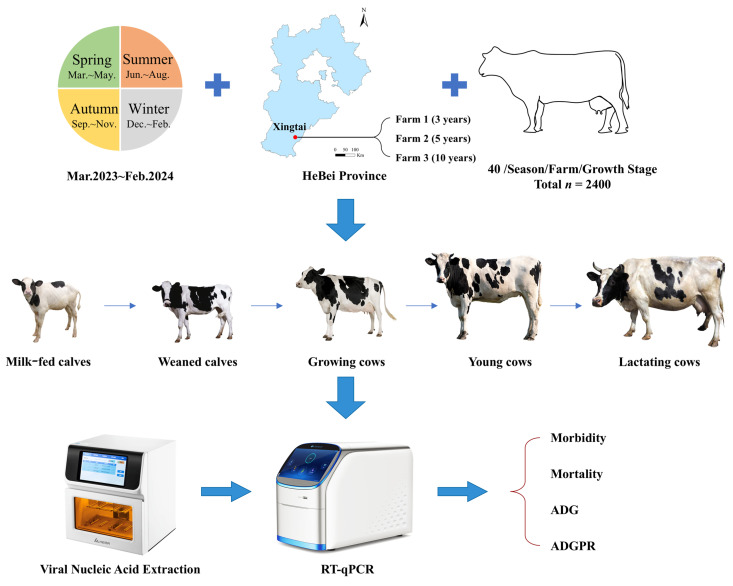
General research ideas.

The research idea is shown in Figure 1.

### 2.1. Sample Collection

The test cows were managed in groups according to growth stages: single-pen feeding (constant temperature at 30 °C) during the neonatal period to ensure colostrum intake ≥ 6 L (IgG ≥ 50 g/L); ad libitum open-feeding during lactation (18% CP); and TMR feeding during the breeding period and adulthood (14–16% CP). Environmental parameters were controlled at 15–25 °C and NH_3_ < 10 ppm throughout the stages, and health data were recorded in real time by electronic ear tags.

From March 2023 to February 2024, a total of 2400 rectal swabs were collected from three large-scale dairy farms in Xingtai, Hebei Province, China. Three representative farms were selected for sample collection, labeled as Farm 1 (3 years old), Farm 2 (5 years old), and Farm 3 (10 years old). The sampling process was conducted randomly, ensuring balanced representation across five growth stages on the basis of age: milk-fed calves (0–2 months), weaned calves (3–6 months), growing cows (7–15 months), young cows (>15 months, nonpregnant), and lactating cows (postpartum). For each growth stage, 40 samples were collected per farm each season (spring: March–May; summer: June–August; autumn: September–November; winter: December–February).

Prior to collection, the perianal area of each cow was disinfected with 75% alcohol. A disposable swab was then gently inserted into the rectum, rotated five times, and placed in a sterile, enzyme-free collection tube containing nucleic acid preservation solution. To prevent degradation of the nucleic acids, the samples were immediately stored in an insulated box with ice packs and later stored at −80 °C for long-term preservation.

### 2.2. Viral Nucleic Acid Extraction

To extract viral nucleic acids, each swab sample was subjected to three freeze–thaw cycles before centrifugation at 12,000 rpm for 5 min. The resulting supernatant was used for nucleic acid extraction. A “Virus RNA Automatic Extraction Kit” (AE3EK013, AniEasy Bio-Technology Co., Ltd., Shenzhen, China) was used at room temperature. A 200 μL aliquot of the supernatant and 5.6 μL of carrier RNA solution were added to each sample well in a deep-well plate. The plate was then placed in an automatic nucleic acid extractor (Zhuhai Hema Medical Instrument Co., Ltd., Zhuhai, China), and the extraction program was followed according to the manufacturer’s instructions. After extraction, the nucleic acids were transferred to 1.5 mL centrifuge tubes and stored at −20 °C for later analysis.

### 2.3. RT–qPCR Analysis

RT–qPCR was used to detect BRV. The BRV RT–qPCR mixture (AE3BRDC003, AniEasy) was thawed on ice and aliquoted into N + 2 fluorescent PCR wells, with 20 μL added to each well. To each reaction well, 5 μL of RNA sample was added, with three technical replicates for each sample. The PCR tubes were then capped, briefly centrifuged at 2000 rpm for 20 s, and placed in the nucleic acid amplification zone. The RT–qPCR system was set to a total reaction volume of 25 μL, with fluorescence signal acquisition during the annealing phase. The FAM fluorophore was used for signal detection, with no quencher. The cycling conditions were as follows: reverse transcription at 45 °C for 10 min, followed by a predenaturation step at 95 °C for 3 min. The amplification phase included denaturation at 95 °C for 10 s and extension at 60 °C for 30 s, which was repeated for 40 cycles. Data analysis was performed after the amplification was complete.

### 2.4. Criteria for Positive Samples

The cycle threshold (Ct) value obtained from RT–qPCR was used to classify the samples. A Ct value ≤ 25 was considered strongly positive, between 25 and 33 was classified as positive, between 33 and 37 was classified as weakly positive, and between 37 and 38.5 was classified as suspected, requiring further testing. A Ct value > 38.5 was classified as negative. If the negative control was amplified, a sample was considered weakly positive if the difference between the Ct values of the negative control and the sample (Ct negative control − Ct sample) was ≥3.

### 2.5. Data Collection for Production Performance

Production performance data, including morbidity, mortality, average daily weight gain (ADG), and average daily weight gain pass rate (ADGPR) data were retrieved from the Intelligent Ranch Management System (Foidn Technology Co., Ltd., Nanjing, China) from March 2023 to February 2024. The system was configured to filter data specifically for milk-fed calves, and the relevant information was exported to Excel for further analysis. Each calf weight data point was recorded by the system at regular intervals via automatic weighing equipment, and these data were used to calculate the ADG. The ADGPR was calculated by determining the proportion of animals whose ADG met the target within a specific time period. Specifically, the ADG of each calf was compared to the target ADG, and the proportion of animals that met or exceeded the target was considered the pass rate. The arithmetic mean of these data points was subsequently calculated, and the results were summarized.

### 2.6. Statistical Analysis

The negative binomial regression analysis was conducted via R (version 4.4.2, R Foundation for Statistical Computing, Vienna, Austria), with R packages including ’MASS’, ’ggplot2’, ’dplyr’, and ’gridExtra’. Prior to conducting the negative binomial regression, necessary data preprocessing was performed, including outliers and others. Categorical variables such as Farm, Season, and Growth Stage were converted to factor types and analyzed via standard statistical models. In the regression analysis, Farm 3 was designated the baseline farm because it was the oldest farm, allowing comparison with the other farms. The impact of each factor was extracted individually, and bar plots were created to visually represent their effects on the BRV positivity rate. Three categorical variables—Farm, Season, and Growth Stage—were included as factor variables. Specifically, ‘Farm’ (e.g., “Farm 1”, “Farm 2”, “Farm 3”) was treated as a factor with “Farm 3” as the reference level; ‘Season’ (spring, summer, autumn, winter) was included with one season as the reference; and ‘Growth Stage’ (e.g., juvenile, adult) was also treated as a factor. This approach allowed for the assessment of each factor’s independent effect on the BRV positive rate. The regression model formula is as follows:

BRV Positive Rate ~ Farm + Season + Growth Stage

Visualization of other results was carried out via GraphPad Prism (version 8.0, GraphPad Software, San Diego, CA, USA). The relationships between the BRV positivity rate and morbidity, mortality, ADG, and ADGPR were analyzed via Spearman’s rho test for correlation and significance (positive correlation, 0 < ρ < 1; negative correlation, −1 < ρ < 0; *, *p* value < 0.05; **, *p* value < 0.01; ***, *p* value < 0.001).

## 3. Results

### 3.1. Prevalence of BRV Across Farms

**Figure 2:**
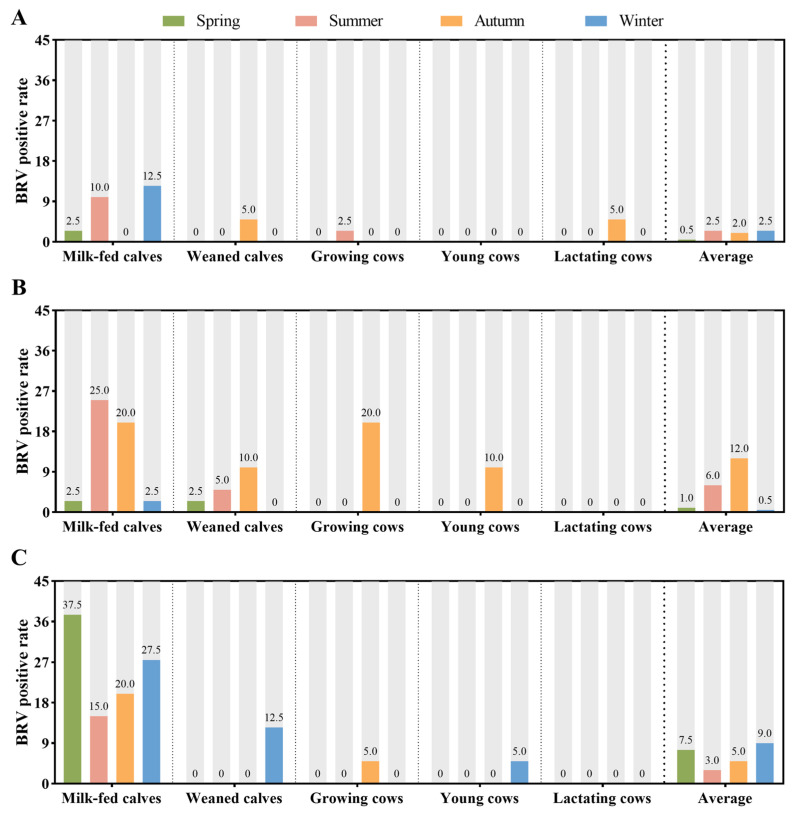
Positivity rates of BRV in dairy cows at different growth stages on Farms 1, 2, and 3 in different seasons. (**A**) Farm 1, (**B**) Farm 2, and (**C**) Farm 3. (*n* = 40 heads/season/farm/growth stage, total *n* = 2400).

On Farm 1 (Figure 2A), milk-fed calves presented the highest prevalence of BRV during the winter (12.5%). The overall positivity rate peaked in the summer and winter, whereas the lowest rates were observed during spring and autumn. For other groups, such as weaned calves, growing cows, and lactating cows, BRV positivity was sporadic, with only occasional detection. Notably, young cows consistently tested negative throughout the study. A clear seasonal trend emerged, with positivity increasing from spring to winter, followed by a decline and subsequent rise, indicating seasonal fluctuations.

Farm 2 (Figure 2B) exhibited a different pattern. BRV was not detected in lactating cows but was detected in the other groups. The highest prevalence occurred in milk-fed calves, with positivity rates of 25.0% in summer and 20.0% in autumn. Positivity in weaned calves gradually increased from 2.5% in spring to 10% in autumn, with no detections in winter. Overall, BRV positivity increased from spring (1.0%) to autumn (12.0%) but declined in winter (0.5%).

On Farm 3 (Figure 2C), the prevalence followed a similar trend to that of Farm 2. BRV was absent in lactating cows, whereas milk-fed calves exhibited a clear seasonal pattern. The prevalence peaked in spring (37.5%), decreased to 15.0% in summer, and increased again in autumn and winter. The annual prevalence was highest in spring (7.5%) and winter (9.0%), with lower rates in summer (3.0%) and autumn (5.0%). A gradual increase in BRV positivity followed a spring decline.

### 3.2. Prevalence of BRV Across Farms, Seasons, and Growth Stages

**Figure 3:**
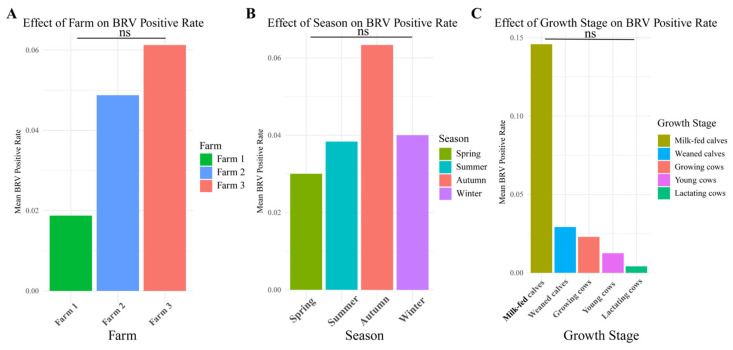
The effects of farm, season, and growth stage on the BRV positive rate were analyzed via a negative binomial regression model. (**A**) Effects of farm type on the BRV positive rate. (**B**) Effects of season on the BRV positive rate. (**C**) Effects of growth stage on the BRV positive rate.

Farm type was a significant factor influencing BRV positivity, with Farm 3 exhibiting the highest positivity rate in milk-fed calves. On the other hand, the highest positivity rates in milk-fed calves on Farm 1 and Farm 2 were relatively low, indicating that the infection pressure or control measures on these younger farms may be more effective (Figure 3A).

Seasonal variation also affected BRV positivity rates, with autumn having the highest prevalence, followed by spring and summer, whereas winter had the lowest prevalence. This trend, as shown in Figure 3B, highlights the need for tailored seasonal management strategies, especially in autumn and spring, when BRV transmission may be more prevalent. The influence of seasonal changes on BRV dynamics warrants additional research into how climate and environmental factors might contribute to increased infection rates.

Finally, the growth stage played a significant role, with milk-fed calves showing the highest BRV positivity rates (Figure 3C).

### 3.3. Correlation Between BRV Positivity and Morbidity in Milk-Fed Calves

**Figure 4:**
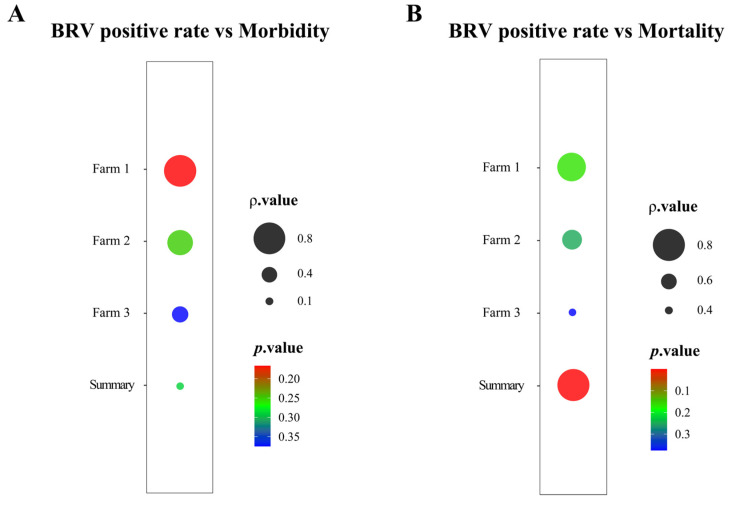
Correlation analysis between BRV positive rate, morbidity, and mortality in milk-fed calves. (**A**) Correlation analysis between BRV positive rate and morbidity; (**B**) correlation analysis between BRV positive rate and mortality.

The mean values of morbidity, mortality, ADG, and ADGPR obtained through the system were calculated, and the results are shown in Table 1. The correlation between BRV positivity and morbidity in milk-fed calves was analyzed across three farms. A strong positive correlation (ρ > 0.5) was observed between these variables in Farms 1 and 2, whereas a weaker correlation (ρ = 0.400) was found in Farm 3. As shown in Figure 4A, the statistical significance of Farms 1 and 2 was not reached (*p* = 0.1667, *p* = 0.2500). This may be due to other confounding factors or inadequate sample size. Figure 4A also illustrates a similar weak correlation in Farm 3 (ρ = 0.4000), which was also not significant (*p* = 0.3750). The summarized data across all three farms, shown in Figure 4A, further diminished the correlation (ρ = 0.1764), with no significant results (*p* = 0.2902).

### 3.4. Correlation Between BRV Positivity and Mortality in Milk-Fed Calves

For the three farms included in the analysis, the correlation between BRV positivity and mortality in milk-fed calves varied. As shown in Figure 4B, Farms 1 and 2 presented a strong positive correlation between BRV positivity and mortality (ρ = 0.8000, ρ = 0.6325), but neither of these correlations reached statistical significance (*p* = 0.1667, *p* = 0.2500). Figure 4B shows that in Farm 3, the correlation was weaker (ρ = 0.4000) and not significant (*p* = 0.3750). However, when the data summarized from all three farms were aggregated, a highly significant positive correlation was observed between BRV positivity and mortality (*p* = 0.0003, ρ = 0.8642). This result suggests that while the relationship between BRV positivity and mortality was not significant for individual farms, the aggregated data revealed a significant positive correlation across the three farms. This highlights the potential impact of BRV infection on mortality in a broader farm context, emphasizing the importance of timely diagnostics and effective management practices to reduce mortality and improve production efficiency.

### 3.5. Negative Correlation Between BRV Positivity and ADG in Milk-Fed Calves

**Figure 5:**
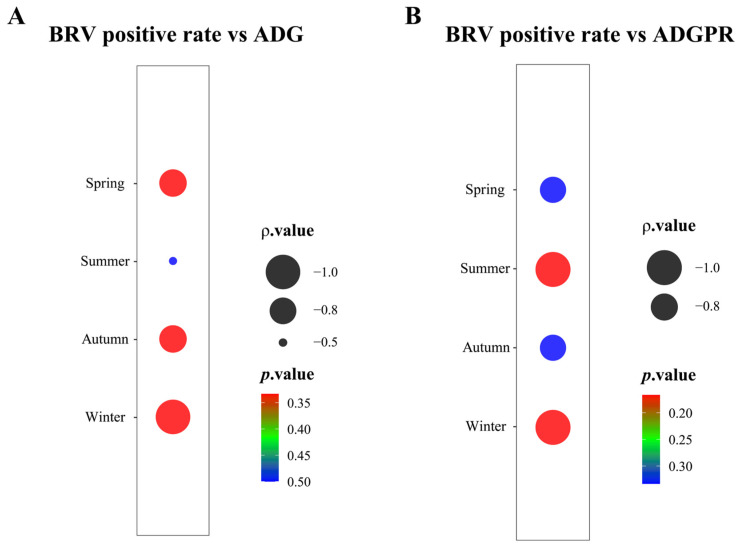
Correlation analysis between BRV positive rate, ADG, and ADGPR in milk-fed calves. (**A**) Correlation analysis between BRV positive rate and ADG; (**B**) correlation analysis between BRV positive rate and ADGPR.

In all four seasons, the relationship between BRV positivity and ADG in milk-fed calves was found to be negative. However, the correlation was not significant in spring, autumn, or winter (*p* = 0.3333, *p* = 0.3333, *p* = 0.1667), with strong negative correlations observed (ρ = −0.8660, ρ = −0.8660, ρ = −1.000) (Figure 5A). Similarly, the results for summer also revealed a nonsignificant negative correlation (*p* = 0.5000, ρ = −0.5000). These findings suggest that although a negative trend exists, statistical significance was not achieved, likely because of factors such as limited sample size, more farms investigated, seasonal variation, or unaccounted external factors such as environmental stress and management practices. In particular, the strong negative correlation in winter requires further investigation, as its lack of significance prevents a clear understanding of its underlying mechanism.

### 3.6. Correlation Between BRV Positivity and the ADGPR in Milk-Fed Calves

In all four seasons, a strong negative correlation was observed between BRV positivity and ADGPR in milk-fed calves. Although this negative correlation was not significant in spring and autumn (*p* = 0.3333) (Figure 5B), it reached −1.000 in summer and winter (*p* = 0.1667), indicating a strong impact of BRV positivity reduction on ADGPR during these seasons. These findings suggest that in summer and winter, controlling BRV infection is crucial for improving calf growth performance. These findings provide important directions for further research, particularly in exploring the impact of other seasonal factors on BRV infection.

## 4. Discussion

This study aimed to assess the prevalence of BRV infection across different growth stages of dairy cows on three farms with varying ages of operation in Xingtai city, Hebei Province, China, from 2023–2024 and to determine its seasonal variation. The results revealed that milk-fed calves were the most commonly infected group, followed by weaned calves. The overall BRV positivity rate in milk-fed calves was 14.58%, whereas that in weaned calves was 8.75%. The annual average BRV positivity rate across all age groups was 4.29%. These findings are consistent with those of previous studies, such as that by Cheng et al. [24], who reported a BRV prevalence of 4.3% (10/233) in diarrheic calves in Northeast China. However, owing to limitations in the present study design, such as the lack of long-term tracking of individual farms and the absence of concurrent testing for other pathogens, such as BVDV, the data presented herein only offer a snapshot of BRV prevalence. Prior to conducting the long-term BRV testing, the dairy cows on the farm were also tested for common diarrhea pathogens, including BVDV and *E coli* K88 and K99. The results of these tests were negative. In light of the aforementioned considerations, the decision was taken to select BRV for the purpose of long-term monitoring. Furthermore, financial constraints were an important limiting factor in the study. Despite these limitations, our study provides valuable insights into the seasonal dynamics of BRV in dairy cows of varying ages and across different farm ages.

In this study, a negative binomial regression model was utilized to analyze the influence of farm, season, and growth stage on BRV positivity rates. The findings indicate that, despite the similarity in management practices and rearing environments across the three farms, a correlation between farm age and BRV positivity is postulated. However, further research is necessary to substantiate this hypothesis. Although this difference did not reach statistical significance, it suggests that farm age may have a potential impact on BRV positivity rates. In terms of seasonal factors, the BRV positivity rate in autumn was significantly higher than that in other seasons, which aligns with the literature indicating that BRV is more prevalent in autumn [25]. Furthermore, analysis of data across five growth stages revealed that milk-fed calves presented significantly higher BRV positivity rates than other stages did, with a noticeable decrease in positivity as age increased. These findings highlight the critical role of milk-fed calves in BRV control, especially during the early stages when their immune systems are not fully developed [26,27]. Overall, this study provides valuable empirical data for understanding the seasonal and growth stage variations in BRV infection and offers theoretical support for the development of vaccination and control strategies.

These results indicate a positive correlation between the BRV positivity rate and both morbidity and mortality in milk-fed calves. Statistical analysis revealed a strong positive correlation between BRV positivity and morbidity and mortality in Farms 1 and 2, suggesting a significant impact of BRV on the health of milk-fed calves on these farms. However, on Farm 3, the correlation between BRV positivity and morbidity and mortality was weaker, possibly because of the different disease prevention strategies or management practices implemented on that farm or the more severe prevalence of other pathogens affecting calf health. Nevertheless, this hypothesis requires further investigation to explore the effects of various management strategies and pathogen exposure on morbidity and mortality.

One of the most concerning findings in this study was the negative impact of BRV on growth performance, particularly ADG, in milk-fed calves. During the winter months, when the negative correlation was most pronounced, the high BRV positivity rate (27.5%) resulted in reduced daily weight gain in the calf group compared to the group with a lower positivity rate (2.5%), with an average daily weight gain of 0.04 kg less. The direct consequence of this was that calves in the high-positivity-rate groups had an average weight deficit of 2.12 kg by 60 days of age and 6.92 kg by 180 days relative to those in the low-positivity-rate groups. This finding is consistent with those of previous studies, such as that by Renaud et al. [3], which reported similar growth retardation in calves infected with bovine coronavirus (BCoV) and *Cryptosporidium parvum*. However, unlike their study, the present research provides a broader perspective by examining the collective impact of BRV across entire groups of calves, which underscores the long-term detrimental effects of BRV infection on farm growth.

Milk-fed calves typically need to increase their body weight from 35–40 kg to 70–80 kg within the first 60 days after birth to complete weaning and enter the weaning period. However, the impact of BRV infection may prolong this process. In the spring, when the BRV incidence increased from 0% to 37.5%, the average daily weight gain compliance rate decreased by 17.25%, resulting in significant economic losses for the farm. On the basis of the actual farming conditions and the present data, we showed that when the BRV positivity rate increased from 0 to 20%, the decrease in ADG could lead to the need for at least an additional 3 days of feeding for the calves to reach the target weight, increasing the cost per calf by approximately USD 10.3–13.75. As the breed size increases, these additional costs could impose a substantial economic burden, highlighting the potential economic losses that BRV infections can cause in the dairy farming industry.

These findings also echo previous research showing that diarrhea, particularly in the context of BRV infection, is a major contributor to growth retardation in calves [2]. Infected calves experience damage to the intestinal epithelium, resulting in villous atrophy and impaired nutrient absorption, which ultimately affects their growth and overall health [28]. The present study examined the impact of BRV on large dairy farms from a large population perspective, investigated the deleterious effects of BRV, and examined the relationship between BRV and key production parameters such as morbidity and mortality. Although the study was limited to the period from 2023–2024 due to certain objective factors, the findings provide valuable data to support the enhancement of BRV control measures on large-scale farms. These data help farms better understand and emphasize the importance and necessity of BRV prevention from a farm-level perspective, highlighting its impact on dairy production performance.

Despite some limitations in this study, such as the inability to track long-term infection status and the lack of concurrent testing for bacterial pathogens, the results provide valuable insights into the seasonal dynamics of BRV infection and its impact on dairy calf health and performance. Future research, particularly long-term monitoring and testing for other potential pathogens, will help provide a more comprehensive understanding of the various factors affecting the health and productivity of dairy farms.

## 5. Conclusions

This study explored the relationships between BRV incidence and farm age, season, and growth stage and its impact on production performance. Farm, autumn, and milk-fed calves were identified as key factors affecting BRV positivity. BRV positivity is correlated with increased morbidity and mortality and decreased ADG/ADGPR, potentially leading to significant economic losses. These findings emphasize the need for targeted control strategies, such as vaccination and improved colostrum management. Despite limitations, such as the lack of long-term cohort analysis, this research provides valuable insights for optimizing BRV control measures to reduce its economic impact on dairy farming.

## Figures and Tables

**Table 1:** Summary table of positive rates of BRV, morbidity, mortality, ADG, and ADWGPR data in milk-fed calves on three farms in different seasons (ADG: average daily weight gain; ADGPR: average daily weight gain pass rate).

Farm	Season	Positive Rate of BRV (%)	Morbidity (%)	Mortality (%)	ADG (kg)	ADGPR (%)
1	Spring	2.50	11.48	1.78	0.98	87.42
	Summer	10.00	12.80	3.51	0.99	90.15
	Autumn	0.00	11.26	1.68	0.99	91.87
	Winter	12.50	12.69	3.38	0.96	91.60
2	Spring	2.50	13.15	3.00	1.00	87.60
	Summer	25.00	25.02	4.64	0.88	88.68
	Autumn	20.00	12.59	2.83	0.95	87.38
	Winter	2.50	11.57	2.51	0.98	94.92
3	Spring	37.50	14.10	4.35	0.94	71.43
	Summer	15.00	12.26	4.00	0.96	90.20
	Autumn	20.00	10.90	3.25	0.95	88.34
	Winter	27.50	11.17	3.93	0.94	91.53

## Data Availability

The data that support the findings of this study are available from the corresponding author, Dewen Tong (e-mail: dwtong@nwsuaf.edu.cn), upon reasonable request.

## Abbreviations

The following abbreviations are used in this manuscript:

MDPI	Multidisciplinary Digital Publishing Institute
DOAJ	Directory of open access journals
TLA	Three-letter acronym
LD	Linear dichroism
BRV	Bovine Rotavirus
ADG	Average daily weight gain
ADGPR	Average daily weight gain pass rate
OECD	Organization for Economic Co-operation and Development
FAO	Food and Agriculture Organization of the United Nations
RT–qPCR	Real-Time Quantitative poly chain reaction
FAM	Carboxyfluorescein
Ct	Cycle threshold
BVDV	Bovine viral diarrhea virus
BCoV	Bovine coronavirus
*E. coli*	*Escherichia coli*
